# Herbal Extract SH003 Suppresses Tumor Growth and Metastasis of MDA-MB-231 Breast Cancer Cells by Inhibiting STAT3-IL-6 Signaling

**DOI:** 10.1155/2014/492173

**Published:** 2014-05-25

**Authors:** Youn Kyung Choi, Sung-Gook Cho, Sang-Mi Woo, Yee Jin Yun, Sunju Park, Yong Cheol Shin, Seong-Gyu Ko

**Affiliations:** ^1^Lab of Clinical Biology and Pharmacogenomics, Department of Preventive Medicine, College of Korean Medicine, Kyung Hee University, Seoul 130-701, Republic of Korea; ^2^Department of Preventive Medicine, College of Korean Medicine, Daejeon University, 62 Daehak-ro, Dong-gu, Daejeon 300-716, Republic of Korea

## Abstract

Cancer inflammation promotes cancer progression, resulting in a high risk of cancer. Here, we demonstrate that our new herbal extract, SH003, suppresses both tumor growth and metastasis of MDA-MB-231 breast cancer cells via inhibiting STAT3-IL-6 signaling path. Our new herbal formula, SH003, mixed extract from *Astragalus membranaceus, Angelica gigas*, and *Trichosanthes kirilowii* Maximowicz, suppressed MDA-MB-231 tumor growth and lung metastasis *in vivo* and reduced the viability and metastatic abilities of MDA-MB-231 cells *in vitro*. Furthermore, SH003 inhibited STAT3 activation, which resulted in a reduction of IL-6 production. Therefore, we conclude that SH003 suppresses highly metastatic breast cancer growth and metastasis by inhibiting STAT3-IL-6 signaling path.

## 1. Introduction

Triple-negative breast cancer (TNBC) constituting 10–20% in breast cancer is highly metastasizing and recurrent with poorer prognoses [1–3]. Although TNBC is sensitive to chemotherapies, TNBC metastases frequently occur and shorten 5-year survival rates of patients [1, 3]. Furthermore, target therapies for TNBC remain yet to be clearly elucidated in clinical trials. Recent studies in cancer therapeutics revisit a traditional herbal medicine, since herbal extracts or mixtures based on the traditional medicines have shown anticancer effects with no or less side effects compared to other anticancer therapeutics including chemical compounds and targeting antibodies [4–6]. Anticancer effects of herbal extracts from* Astragalus membranaceus* (Am),* Angelica gigas* (Ag), or* Trichosanthes Kirilowii *Maximowicz (Tk) have been revealed in different cancer cell types such as leukemia, hepatocellular carcinoma, colon cancer, non-small-cell lung cancer, and gastric cancer cells [7–13]. Furthermore, extracts from a mixture of Am and Ag have been shown to affect various diseases including hematologic diseases or endocrine disorders [14–16].

Inflammation is a risk factor in cancer disease [17–22], which is tightly linked to cancer progression including tumorigenesis and metastasis [23, 24]. Cancer inflammation is activated by several inflammatory cytokines such as TNF-*α*, IL-1, IL-6, IL-8, and IL-18 [25]. Particularly, IL-6 as a poor prognostic factor in breast cancer patients progresses cancer metastasis [26]. In addition, IL-6-induced dimerization of IL-6 receptor activates STAT3, which contributes to cancer progression in cancer inflammatory environment [27, 28]. Recent studies have shown that STAT3 activation leads to TNBC progression, suggesting that STAT3 is likely to be a therapeutic target for TNBC [29, 30].

On the basis of the traditional medicine, SH003 was extracted from the herbal mixtures of Am, Ag, and Tk. SH003 showed anticancer effects on different breast cancer cells without affecting normal epithelial cell viability, both* in vitro* and* in vivo*. Moreover, SH003 suppresses MDA-MB-231 growth and metastasis by inhibiting STAT3-IL-6 pathway, thereby suggesting that SH003 may be useful for treating TNBC.

## 2. Materials and Methods

### 2.1. Reagents, Preparation of SH003, and Cell Lines

SH003 consists of Am, Ag, and Tk, which is based on the principle of the traditional medicine. All extracts were provided from Hanpoong Pharm and Foods Company (Jeonju, Republic of Korea) manufactured by the Good Manufacturing Product (GMP). Dried extracts were dissolved in 30% ethanol to prepare a stock solution of 20 mg/mL. The stock solution was stored at −80°C. HPLC and UPLC were performed to confirm characteristics of herbal mixtures including each component (Hanpoong Pharm and Foods Company). Breast cancer cell lines, MCF-7 (hormone-positive), T47D (hormone-positive), SKBR-3 (HER-2-positive), BT-20 (TNBC, noninvasive), and MDA-MB-231 (TNBC, highly metastatic) were cultured in DMEM medium with 10% fetal bovine serum and 1% antibiotics. Rat normal intestinal epithelial cells (RIEs) were also cultured in the same condition as above. GBL-60 cells (kindly provided by Dr. Sun Ha Paek at Seoul National University Hospital, Seoul, Republic of Korea) isolated from the brain of a patient who suffered from brain-metastasized breast cancer were also cultured in DMEM, which was approved by an Institutional Review Board at the Seoul National University Hospital [31].

### 2.2. Cell Viability Assay and Flow Cytometry

Cells were seeded on 96-well plates and treated with different herbal extracts for 24 hours to 72 hours. Cell viability was measured by MTT assays. Absorbance was read at 570 nm on the ELISA reader (Molecular Devices, Palo Alto, CA, USA). Cells were seeded in 6-well plates and treated with each extract for 24 hours. Cells were then harvested and stained with propidium iodide (PI, 50 *μ*g/mL) at room temperature in the dark. PI-positive cells were detected using FACSCalibur (BD Biosciences, San Jose, CA, USA).

### 2.3. Cell Migration, Invasion Assay, and Anchorage-Independent Assay

Cell migration was measured by scratching assays. Cells were seeded in 6-well plates and then scratched. 24 hours after treatments with herbal extracts, migrated cell numbers were counted. For invasion assays, cells were cultured in the upper chambers precoated with matrigels and treated with each extract for 24 hours. After swapping the upper chamber carefully, invaded cell numbers in four fields randomly chosen were counted. For anchorage-independent assays, cells were cultured on soft agar plates and treated with extracts every second day. At day 15, cells were stained with 0.5% crystal violet to be visualized and colonies were counted with photomicroscope.

### 2.4. Western Blot and Immunofluorescence Assays

Cells were lyzed with RIPA buffer and total 30 *μ*g of protein was loaded on 6–12% SDS-PAGE. After transferring to PVDF membranes, each membrane was blotted with the appropriate antibodies. Anti-PARP, -p-EGFR, -EGFR, -p-STAT3, -STAT3, -p-JAK1, -p-JAK2, -p-AKT, and -AKT antibodies were purchased from Cell Signaling (Danvers, MA, USA). Anti-p-SRC, -SRC, -p-ERK1/2, -ERK1/2, -VEGF, -Cyclin D, -MMP-9, -Survivin, and -Tubulin were purchased from Santa Cruz Biotechnology (Santa Cruz, CA, USA). Immunofluorescence assays for p-STAT3 nuclear translocation in MDA-MB-231 cells were done with anti-p-STAT3 antibody and anti-Alexa Fluor-488 antibody (Invitrogen, Eugene, OR, USA). For the counter staining, TOPRO-3 (Invitrogen, Eugene, OR, USA) was used to stain the nucleus. Images were obtained with Olympus FV10i Self-Contained Confocal Laser System.

### 2.5. Luciferase Assay

Luciferase assays were performed with the dual luciferase assay kits (Promega, Madison, WI, USA) according to the manufacturer's instructions. In brief, p4xM67-TK-luc plasmid (Addgene plasmid 8688, Addgene, Cambridge, MA, USA) [32] containing four copies of the STAT-binding site (TTCCCGTAA) was transfected in 293T or MDA-MB-231 cells and then extracts were treated for 24 hours. EF.STAT3C.UBC.GFP and EF.STAT3DN.UBC.GFP (Addgene plasmids 24983 and 24984, Addgene, Cambridge, MA, USA) [33] were transfected into 293T or MDA-MB-231 cells, which were subjected to the luciferase assays. Luciferase assays were conducted in quadruplicate and independently repeated at least three times. Representative data were described as means ± standard deviations. For knockdown strategies, pSIH1-puro-STAT3 shRNA (Addgene plasmid 26596, Addgene, Cambridge, MA, USA) [34] was used.

### 2.6. Real-Time PCR, Chromatin Immunoprecipitation Assays, and ELISA

Total RNAs were extracted with Trizol (Invitrogen, NY, USA). After measuring the RNA concentration by using the NanoDrop ND-1000 spectrophotometer, 1 *μ*g of total RNA was reverse-transcribed using cDNA synthesis kit (TaKaRa, Kusatsu, Shiga, Japan).* GAPDH* was used for an internal control. Primers used are as follows: 5′-AATCCCATCACCATCTTCCA-3′ (*GAPDH* F), 5′-TGGACTCCACGACGTACTCA-3′ (*GAPDH* R), 5′-AACCTTCCAAAGATGGCTGAA-3′ (*IL-6* F), and 5′-CAGGAACTGGATCAGGACTTT-3′ (*IL-6* R). Quantitative real-time PCRs were performed using SYBR green Master Mix (Takara, Shiga, Japan) in LightCycler 480 (Roche, Switzerland). Chromatin immunoprecipitation (ChIP) assays were performed using EpiSeeker ChIP kit (Abcam, Cambridge, UK) according to the manufacturer's instructions. In brief, cells were treated with SH003 for 3 hours and then fixed with 0.75% formaldehyde. Lysates were then sonicated and immunoprecipitated with anti-STAT3 antibody (Cell Signaling, Danvers, MA, USA). After reverse cross-linking, immunoprecipitated and purified DNA fragments were subjected to real-time PCRs. STAT3 binding region (−143 bp~48 bp) was amplified using primers as follows: F: 5′-GTTGTGTCTTGCCATGCTAAAG-3′, R: 5′-AGAATGAGCCTCAGACATCTCC-3′. ELISAs were performed with human IL-6 ELISA kit (BD Biosciences,San Jose CA, USA) according to the manufacturer's instructions.

### 2.7. *In Vivo* Studies

Animal studies were approved by Kyung Hee University Institutional Animal Care and Use Committee (KHU-IACUC). Six-week-old nude (*Nu/Nu*) mice were purchased from Oriental Science and injected s.c. with 1 × 10^6^ MDA-MB-231 cells. When tumor volume reached 50 mm^3^, mice were randomly grouped and extracts were p.o. added daily. Body weights and tumor volumes were measured three times a week. At the end of experiments, mice were sacrificed and all organs including tumors were fixed with 4% formaldehyde. Blood was also taken from the heart and subjected to the blood test. Lung metastasis was measured by counting metastatic colony numbers on lungs. Fixed organs were embedded in paraffin and stained with hematoxylin and eosin for histological observations. Immunohistochemistry was performed with anti-CD31 antibody (Abcam, Cambridge, UK).

### 2.8. Statistics

Data were presented as means and standard deviations. *P* values less than 0.05 in the two-tailed Student's* t*-test were considered statistically significant.

## 3. Results

### 3.1. HPLC Analysis of SH003

**Figure 1:**
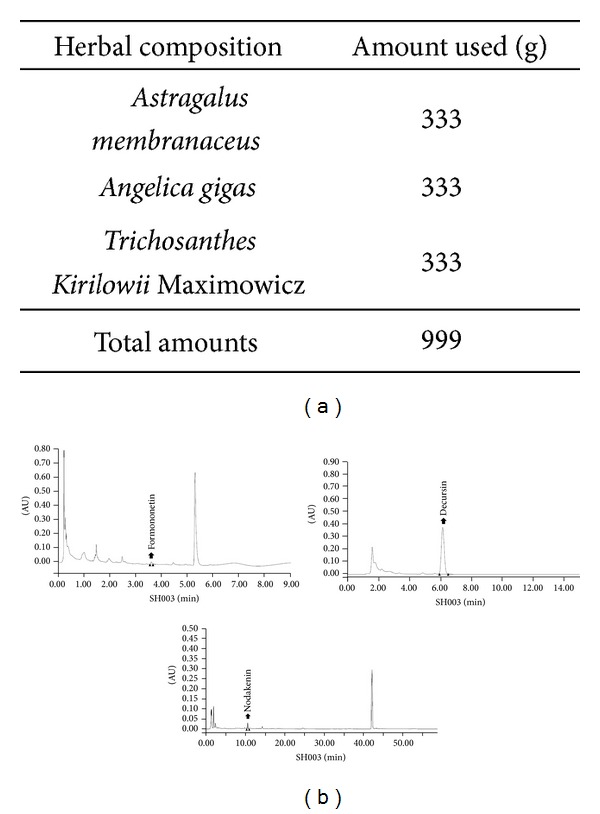
HPLC profile of SH003. (a) Composition of SH003. (b) HPLC identification of components in SH003. Formononetin, decursin, and nodakenin were detected in Am and Ag. Three components in SH003 were detected at 3.6 min, 6.1 min, and 11.0 min.

SH003 was extracted from the mixture of three different herbs (Figure 1(a)). A characterization of SH003 was based on retention times and UV spectra of standard chemicals at wavelengths of 260 nm (formononetin), 280 nm (decursin), and 330 nm (nodakenin): formononetin (3.6 min) for Am, decursin (6.1 min) for Ag, and nodakenin (11.0 min) for Ag (Figure 1(b)). However, we failed to detect an index compound for Tk. We assumed that technical limitations might cause that failure.

### 3.2. SH003 Inhibits MDA-MB-231 Tumor Growth and Metastasis* In Vivo*

**Figure 2:**
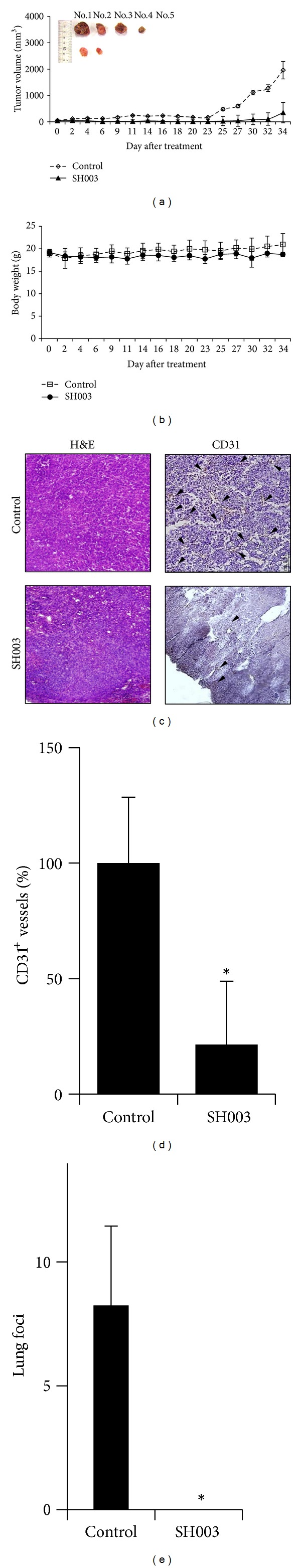
SH003 suppresses tumor growth* in vivo*. (a) 1 × 10^6^ MDA-MB-231 cells were s.c. injected and nude mice (*n* = 5/group) were p.o. administrated with the indicatives until 34 days. Xenograft tumor volumes were measured three times a week by a caliper. *P < 0.05. (b) Body weights were measured three times a week. (c) Tumor tissues were stained with hematoxylin and eosin. Photo images were taken at 20x magnification. Tumor tissues were also stained with anti-CD31 antibody to detect tumor angiogenic vessels. The bar indicates 10 *μ*m. (d) To measure tumor angiogenic vessels in tumor cohorts, CD31-positive vessels were counted. *P < 0.05. (e) Pulmonary metastases were determined by counting foci at lungs.

To examine anticancer effects of SH003 on MDA-MB-231 cells* in vivo*, we performed the xenograft mouse tumor growth assays. When mice were orally administrated with SH003 (500 mg/kg) every day and sacrificed at day 34 posttreatment, extracts repressed tumor growth. Average tumor volumes of control (*n* = 4) and SH003 (*n* = 5) at day 34 were approximately 1958.74 mm^3^ and 348.164 mm^3^, respectively (Figure 2(a)). In addition, SH003 did not affect body weights of mice until 34 days (Figure 2(b)).

When tumor tissues were stained with hematoxylin and eosin, we found that tumor cohort treated with SH003, compared to that with control, was well differentiated (Figure 2(c)). Tumor tissues were then stained with anti-CD31 antibodies to detect tumor vessels because tumor angiogenesis is a bridge for distant metastasis [35]. SH003 compared to the control reduced vessel numbers in tumor burdens by approximately 79% (Figures 2(c) and 2(d)). Thus, our data indicate that SH003 inhibits tumor growth.

Next, we conducted* in vivo* experimental metastasis assays to examine SH003 effect on a distant metastasis. When metastatic tumor colonies on lungs were counted, SH003 compared to control strongly reduced colony numbers by approximately 100% (Figure 2(e)). Thus, our data indicate that SH003 inhibits MDA-MB-231 tumor growth and metastasis,* in vivo*.

### 3.3. SH003 Inhibits Cell Proliferation and Induces Apoptosis

**Figure 3:**
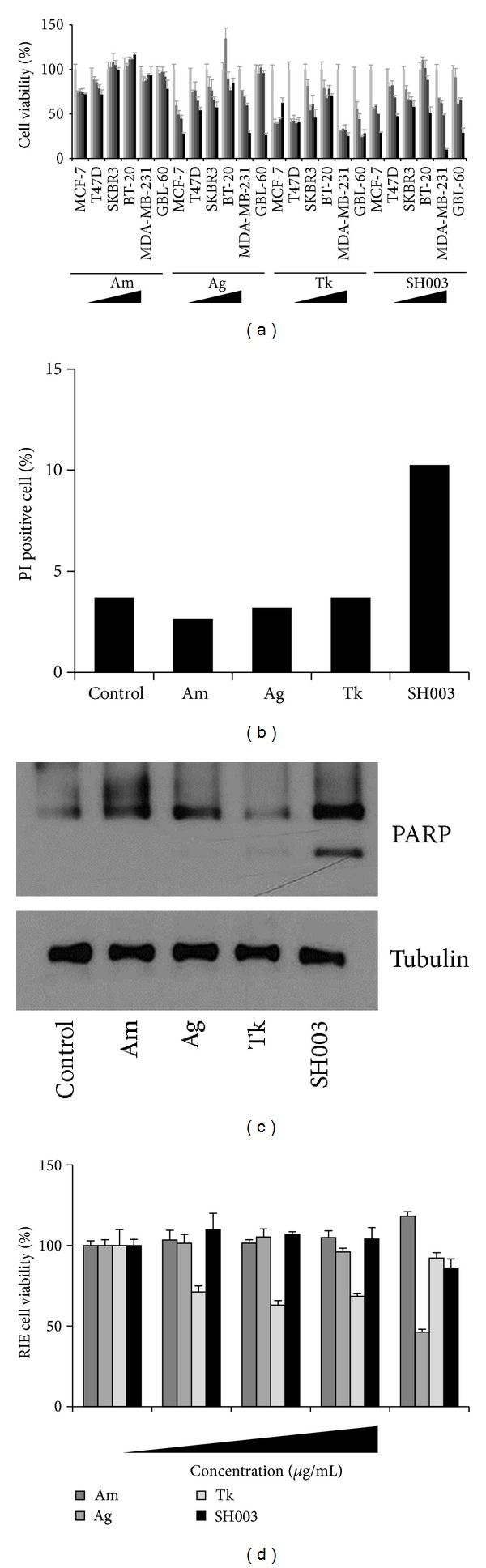
SH003 inhibits MDA-MB-231 growth and induces apoptosis. (a) Different breast cancer cells were seeded on 96-well plates and treated with each extract at different concentrations for 72 hours. Experiments were performed three times in sextuplicate. Representative data were presented as the means and standard deviations. Right triangles indicate the doses of each extract (0, 50, 100, 200, and 500 *μ*g/mL), which was also marked with bars in different colors. (b) MDA-MB-231 cells were treated with 500 *μ*g/mL of the each extract. Cells were stained with propidium iodide (PI, 50 *μ*g/mL) at room temperature in the dark. PI-positive apoptotic cells were detected using FACSCalibur. *P < 0.05. (c) MDA-MB-231 cells were treated with the indicatives at 500 *μ*g/mL for 24 hours and then subjected to western blots. Tubulin was used for the intimal control. (d) RIE cells were seeded on 96-well plates and treated with each extract at different concentrations for 72 hours. Experiments were performed three times in sextuplicate. Representative data were presented as the means and standard deviations.

To examine anticancer effects of SH003 on different types of breast cancer cells, MCF-7, T47D, SKBR-3, BT-20, MDA-MB-231, and GBL-60 cells were treated with different doses of each component of SH003 for 72 hours. While all herbal extracts we tested affected viabilities on different breast cancer cell lines, SH003 much strongly inhibited MDA-MB-231 cell viability at 500 *μ*g/mL. When MDA-MB-231 cells were treated with SH003 at 500 *μ*g/mL for 72 hours, percentages of viable MDA-MB-231 cells were approximately 9.8% (Figure 3(a)). In addition, SH003 highly increased PI-positive apoptotic cell numbers (Figure 3(b)). Accordingly, SH003 caused PARP cleavages, whereas single components did not affect it (Figure 3(c)). In addition, SH003 did not affect normal rat intestinal epithelial cell viabilities, while an extract from either Ag or Tk reduced cell viability (Figure 3(d)). Those indicate that SH003 ameliorates adverse effects of each component of SH003. Thus, our data indicate that SH003 but not each component uniquely inhibits MDA-MB-231 cell proliferation via apoptosis without affecting normal cell viability.

### 3.4. SH003 Inhibits Cell Proliferation, Migration, Invasion, and Anchorage-Independent Growth

**Figure 4:**
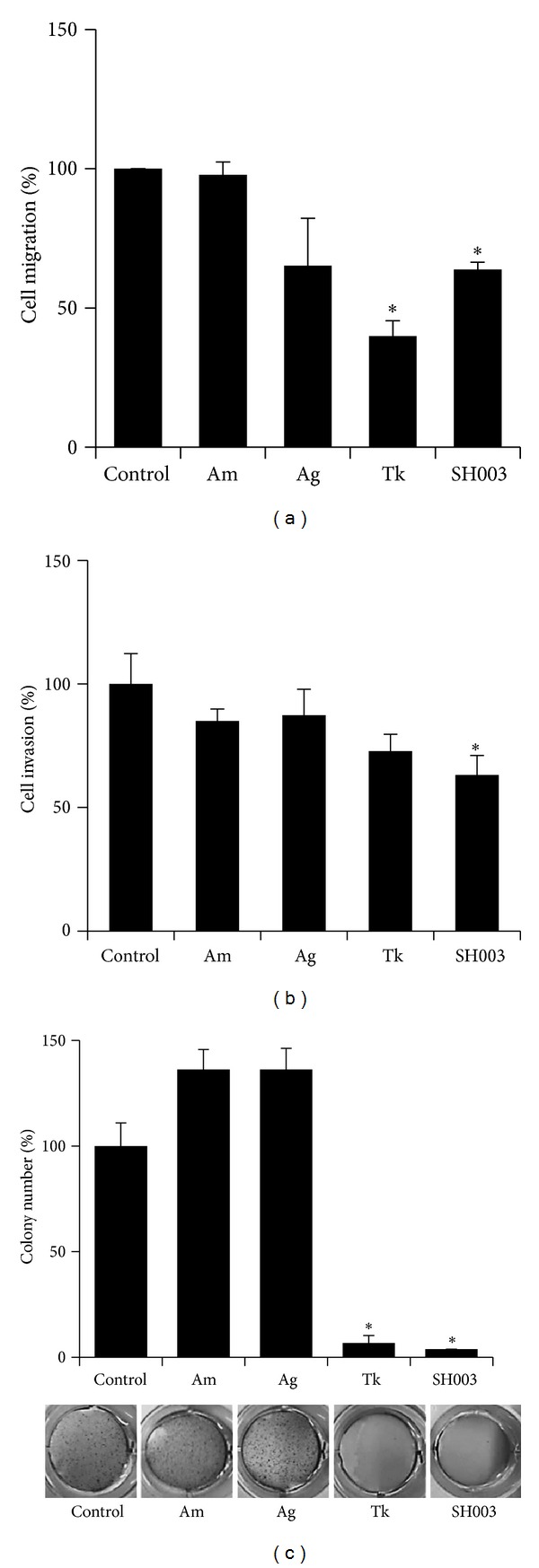
SH003 inhibits metastatic abilities* in vitro*. (a) MDA-MB-231 cells were scratched and treated with the indicatives for 24 hours. Cell migration was determined by counting cell numbers migrated from the wounding region. *P < 0.05. (b) MDA-MB-231 cells were cultured on the upper chambers and treated with the indicatives for 24 hours. Invading cells were stained with crystal violet and then cell numbers were measured. *P < 0.05. (c) MDA-MB-231 cells were cultured in soft agars and treated with the indicatives for 15 days. Colonies were then stained with crystal violet. *P < 0.05.

We next examined whether SH003 affects migratory abilities of MDA-MB-231 cells. 50 *μ*g/mL of SH003 inhibited MDA-MB-231 cell migration by approximately 40% (Figure 4(a)). When we examined an invasiveness of MDA-MB-231 cells, SH003 at 50 *μ*g/mL inhibited cell invasion by 30% (Figure 4(b)). Next, in the soft agar assays, SH003 at 500 *μ*g/mL inhibited anchorage-independent growth of MDA-MB-231 by 95% (Figure 4(c)). Thus, our data indicate that SH003 inhibits* in vitro* metastatic abilities of MDA-MB-231 cells such as cell migration, invasion, and anchorage-independent growth.

### 3.5. SH003 Inhibits EGFR-SRC-STAT3 Phosphorylation and STAT3 Transcriptional Activation

**Figure 5:**
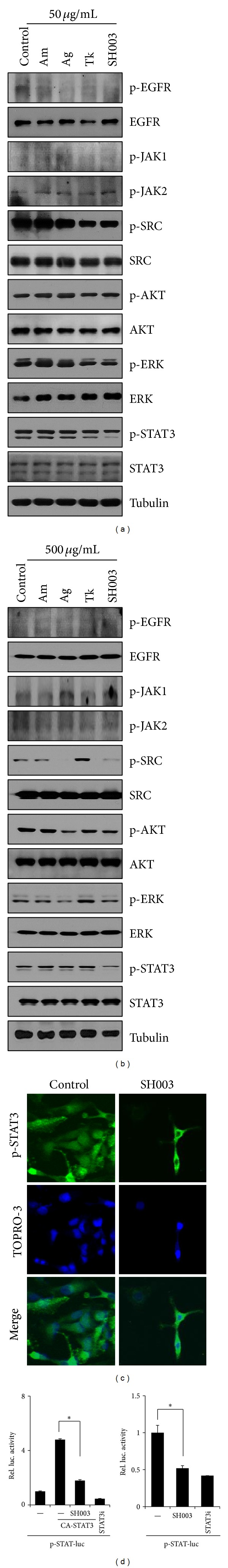
SH003 selectively inhibits STAT3 phosphorylation and transcriptional activity. ((a) and (b)) MDA-MB-231 cells were treated with the indicatives at 50 or 500 *μ*g/mL for 15 minutes and then subjected to western blots with the antibodies indicated. Tubulin was used for the internal control. (c) Cells were treated with the indicatives for 6 hours and then stained with anti-p-STAT3 antibody (green) and TOPRO-3 (blue). 20x objectives. A scale bar indicates 10 *μ*m. (d) Representative data for the luciferase assays. 293T (left) and MDA-MB-231 (right) cells were transfected with the indicatives and then treated with each extract for 24 hours. Experiments were performed in triplicate. Bars indicate means and standard deviations. *P < 0.05.

To decipher anticancer effects of SH003 on MDA-MB-231 cells, we next examined intracellular signaling pathway. Cells were treated with each extract at 50 *μ*g/mL (Figure 5(a)) or 500 *μ*g/mL (Figure 5(b)) for 15 minutes and subjected to the western blots. While phosphorylation of EGFR and SRC was partly reduced by 50 *μ*g/mL of SH003 or each component (Am, Ag, and Tk), STAT3 phosphorylation was strongly and selectively inhibited by SH003. Furthermore, STAT3 phosphorylation was also selectively inhibited by SH003 at 500 *μ*g/mL, while each component at 500 *μ*g/mL did not repress it. Therefore, we assumed that SH003 selectively blocked STAT3 phosphorylation.

Next, we examined whether SH003 affects transcriptional activities of STAT3. When STAT3 nuclear translocation was examined, SH003 at 500 *μ*g/mL blocked nuclear translocation of phosphorylated STAT3 (Figure 5(c)). In the luciferase assays, SH003 at 500 *μ*g/mL also inhibited transcriptional activities of STAT3 in constitutively active STAT3- (CA-STAT3-) overexpressed 293T cells, while STAT3 silencing (STAT3i) in 293T cells reduced STAT3-dependent transcriptional activities (Figure 5(d), left). Likewise, SH003 reduced STAT3 transcriptional activities in MDA-MB-231 cells where STAT3 is constitutively activated, which was similar to the effect of STAT3 silencing on STAT3 transcriptional activity (Figure 5(d), right). Therefore, our data indicate that SH003 selectively inhibits STAT3 activity.

### 3.6. SH003 Inhibits Expression of STAT3 Target Genes and IL-6 Production

**Figure 6:**
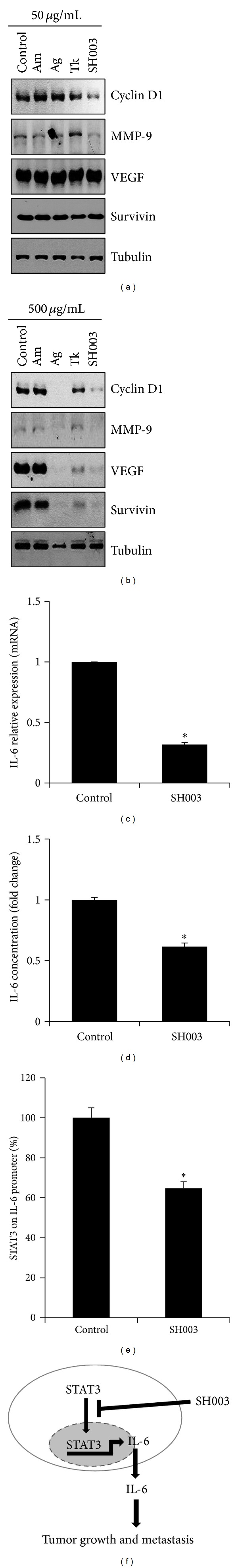
SH003 inhibits STAT3 target gene expression. ((a) and (b)) MDA-MB-231 cells were treated with the indicatives at 50 or 500 *μ*g/mL for 24 hours and then subjected to western blots with the antibodies indicated. Tubulin was detected as a loading control. (c) MDA-MB-231 cells were treated with the indicatives at 500 *μ*g/mL for 24 hours and then subjected to real-time PCR for IL-6 mRNA expression levels. Experiments were performed in triplicate. Bars indicate means and standard deviations. *P < 0.05. (d) MDA-MB-231 cells were treated with the indicatives at 500 *μ*g/mL for 24 hours and then harvested culture media. IL-6 levels were analyzed with ELISA assay. Experiments were performed in triplicate. Bars indicate means and standard deviations. *P < 0.05. (e) Cells were treated with SH003 for 6 hours and then subjected to chromatin immunoprecipitation assays to test STAT3 interaction with IL-6 promoter. (f) A schematic model for anti-TNBC roles of SH003. TNBC has highly metastatic characteristics with constitutively active STAT3. SH003 selectively targets STAT3-dependent IL-6 production, resulting in the inhibition of TNBC growth and metastasis.

As SH003 suppressed STAT3 activation, we next examined whether SH003 affects expression patterns of STAT3-dependent genes. SH003 at 500 *μ*g/mL inhibited protein expression levels of STAT3-dependent genes such as Cyclin D, MMP-9, VEGF, and Survivin, while 50 *μ*g/mL of SH003 only decreased levels of Cyclin D1 and MMP-9 (Figures 6(c) and 6(d)). Those data indicated that expression patterns of those genes might be restricted by STAT3 transcriptional activity and that SH003 effect on those genes was not selective. As shown in Figure 6(c), we found that SH003 at 50 *μ*g/mL or 500 *μ*g/mL decreased IL-6 mRNA level by approximately 65% and 68%, respectively. Next, when MDA-MB-231 cells were treated with SH003 at 50 *μ*g/mL or 500 *μ*g/mL, their cultured media were subjected to ELISA assays. SH003 significantly inhibited secreted IL-6 level by approximately 33.5% and 38.6%, respectively (Figure 6(d)). To confirm if SH003 inhibits STAT3 transcriptional activity for IL-6 expression, we performed chromatin immunoprecipitation assays. When MDA-MB-231 cells were treated with SH003 at 50 *μ*g/mL or 500 *μ*g/mL for 6 hours, SH003 significantly blocked STAT3 interaction with IL-6 promoter region (Figure 6(e)). Thus, our data suggest that SH003 selectively inhibits STAT3-dependent IL-6 expression (Figure 6(f)).

## 4. Discussion 

TNBC is highly metastasizing with a severe recurrence rate, causing a death of patients [1–3, 36–38]. Nevertheless, TNBC is yet clearly curable. Traditional herbal medicines are revisited in cancer biology because those have less adverse effects but better anticancer effects [4, 5]. In this study, we found that SH003 strongly suppressed tumor growth and metastasis of MDA-MB-231 cells defined as TNBC by inhibiting STAT3 activity. Thus, our new herbal extract SH003 appears to be useful for TNBC treatment.

SH003 is extracted from the mixture of Am, Ag, and Tk. Our* in vitro* studies demonstrate that the extract from either Ag or Tk is highly toxic in normal intestinal epithelial cells, while our data and previous reports have shown that the extract from Am, Ag, or Tk inhibited cancer cell growth [7, 10–13]. However, SH003 ameliorated this adverse effect and effectively inhibited tumor growth and metastatic abilities of MDA-MB-231, highly metastatic TNBC cell line,* in vitro*. Furthermore, SH003 suppressed* in vivo* MDA-MB-231 growth and metastasis with no effect on body weights. Thus, SH003 is safe and effective, both* in vivo *and* in vitro*.

STAT3 is crucial for cancer development and metastasis as well as cancer inflammation [39–43] and frequently activated in different types of cancers such as breast, lung, renal, prostate, pancreatic, colon, gastric, cervical, and ovarian cancers [44–47]. SH003 inhibited STAT3 transcriptional activity, while each component did not affect it. Interestingly, 50 *μ*g/mL of SH003 reduced expression levels of MMP-9 and Cyclin D1 with no alterations of Survivin and VEGF, whereas 500 *μ*g/mL of SH003 reduced all we tested. Furthermore, each component also reduced protein expression of those genes. As SH003 uniquely inhibited STAT3-dependent IL-6 expression, our data suggest that SH003 may selectively target STAT3-IL-6 pathway. Meanwhile, we could not exclude a possibility that SH003 is likely to target other molecules beyond STAT3 to suppress MDA-MB-231 cell growth and metastatic abilities. In addition, it remains to be defined how SH003 has this selective effect.

## 5. Conclusions

In conclusion, we provide evidence that anticancer effect of SH003 on MDA-MB-231 cells results from the inhibition of STAT3-dependent IL-6 production. As STAT3 is mutated in different cancer types, it is worth testing if SH003 is able to target those types of cancer cells.
